# Stress in Adolescents with a Chronically Ill Parent: Inspiration from Rolland’s Family Systems-Illness Model

**DOI:** 10.1007/s10882-012-9291-3

**Published:** 2012-07-06

**Authors:** D. S. Sieh, A. L. C. Dikkers, J. M. A. Visser-Meily, A. M. Meijer

**Affiliations:** 1 Research Institute of Child Development and Education, University of Amsterdam, the Netherlands, Nieuwe Prinsengracht 130, 1018 VZ Amsterdam, the Netherlands; 2 Centre of Excellence in Rehabilitation Medicine, Rehabilitation Centre De Hoogstraat, Rembrandtkade 10, 3583 TM Utrecht, the Netherlands; 3 Department of Education, University of Amsterdam, Nieuwe Prinsengracht 130, 1018 VZ Amsterdam, the Netherlands

**Keywords:** Chronic medical condition, Adolescent, Stress, Family-systems illness model

## Abstract

This article was inspired by Rolland’s Family Systems-Illness (FSI) model, aiming to predict adolescent stress as a function of parental illness type. Ninety-nine parents with a chronic medical condition, 82 partners, and 158 adolescent children (51 % girls; mean age = 15.1 years) participated in this Dutch study. The Dutch Stress Questionnaire for Children was used to measure child report of stress. Ill parents completed the Beck Depression Inventory. Children filled in a scale of the Inventory of Parent and Peer Attachment measuring the quality of parent attachment. Both parents filled in the Parent-Child-Interaction Questionnaire-Revised. We conducted multilevel regression analyses including illness type, the ill parent’s depressive symptoms, family functioning (quality of marital relationship, parent-child interaction, and parent attachment), and adolescents’ gender and age. Four regression analyses were performed separately for each illness type as defined by disability (Model 1), and onset (Model 2), course (Model 3), and outcome of illness (Model 4). In all models, higher adolescent stress scores were linked to lower quality of parent-child interaction and parent attachment, and adolescents’ female gender. The four models explained approximately 37 % of the variance in adolescent stress between individuals and 43-44 % of the variance in adolescent stress between families. Adolescent stress was not related to parental illness type. Our results partially supported the FSI model stating that family functioning is essential in point of child adjustment to parental illness. In the chronic stage of parental illness, adolescent stress does not seem to vary depending on illness type.

## Introduction

Children of parents with a chronic medical condition (CMC) are at an increased risk for developing health-related and social-emotional problems, such as somatic complaints, social isolation, and excessive concern to acquire an illness themselves (Compas, 1994; Earley and Cushway 2002; Faulkner and Davey 2002; Pedersen and Revenson 2005). Also more recent evidence suggests that these children show internalizing problems (e.g., anxiety and depressed mood) and externalizing problems, that is, aggressive and rule-breaking behavior (Ivarsson et al. 2002; Sieh et al. 2010a). Children with parental CMC also display elevated stress levels (Pedersen and Revenson 2005; Sieh et al. 2010b). Stress in children presumably acts as a mediator between illness-related factors and child outcomes, and it is therefore an important variable to examine (Pakenham and Bursnall 2006; Pedersen and Revenson 2005). We choose adolescents as target group because they seem to be especially vulnerable to developmental problems and may adopt more caregiving responsibilities than latency-aged children (Korneluk and Lee 1998; Sieh et al. 2010a). Hence, this study will focus on adolescent stress.

According to the Family Systems-Illness (FSI) model of Rolland (1987, 1999), adolescent stress depends on specific illness-related factors, resulting in a differentiation into illness type depending on the diagnosis (Compas et al. 1994; Sieh et al. 2010a). The FSI model classifies CMC’s as a function of illness type and family functioning. Illness type can be categorized based on the presence of disability (non-disabling versus disabling), its onset (gradual versus acute), its course (progressive, episodic, or constant), its outcome (fatal versus non-fatal), and its time stage (beginning versus terminal). Disability implicates problems with activities of daily living, and communicative and cognitive abilities. Disability of parents appears to be associated with elevated stress levels in the family (Pedersen and Revenson 2005; Rolland 1987, 1999; Verhaeghe et al. 2005). Concerning onset, diseases can have an acute onset, forcing the family to adapt in a short period of time, which often causes high stress levels directly after the diagnosis. Conversely, diseases can be qualified by a gradual onset, requiring continuous adjustment. The course of a chronic illness varies depending on the diagnosis and may be categorized based on the pattern of expected trajectory. A progressive illness increases in severity, inducing cumulative responsibilities for family caretakers over time, which is related to elevated stress levels in family members. An episodic illness is represented by exacerbations and remissions that require flexibility of all family members. Constant illnesses are often marked by an acute onset, implicating that the amount of illness-related stressors remains the same after recovery (Pedersen and Revenson 2005; Rolland 1987, 1999). Finally, the illness outcome is characterized by the possibility of death or shortened life span versus non-fatal conditions. The most important element is the initial doubt whether a disease will cause death (Rolland and Williams 2005). It is crucial to add that the FSI model considers illness type in relation to the illness stage (crisis, chronic stage, and terminal stage) and, in turn, in relation to family functioning. Accordingly, the stressors associated with the crisis stage of a less severe condition could easily generate more strain on a family system than the chronic phase of a more serious condition.

It is well-documented that CMC has a tremendous impact on psychological characteristics of ill parents, especially in terms of an increased risk for depression (Visser-Meily and Meijer 2006). For example, in the chronic stage of stroke, depression is a hidden issue that is linked to disability in parents (Van de Port et al. 2007). Similarly, a longitudinal study on children of stroke patients ascertained that depressive symptoms of ill parents predict long-term stress in children (Sieh et al. 2010b). Parental depression is often accompanied by decreased emotional availability of the parent and may directly and indirectly affect the quality of family relationships (Faulkner and Davey 2002). This study also takes the level of ill parents’ depressive symptoms into account because of the strong association with family functioning.

The FSI model proposes that adolescent stress is associated with family functioning (Rolland 1987, 1999; Rolland and Williams 2005). In this study, family functioning is conceptualized by means of three interpersonal variables: quality of marital relationship, quality of parent-child interaction, and quality of parent attachment. Previous research revealed that the quality of marital relationship decreases following parental stroke. Two months after discharge from the rehabilitation center, it correlated with long-term stress in children (Sieh et al. 2010b). Researchers are inconclusive whether the relationship between quality of marital relationship and adolescent functioning is direct or bidirectional (Compas et al. 1994; Compas et al. 1996; Faulkner and Davey 2002; Sieh et al. 2010b; Veach 1999). However, they reached unanimity that the parent-child relationship should be evaluated by both parent and adolescent as the interpretations of this relationship might differ depending on the informant (Korneluk and Lee 1998; Hocking and Lochman 2005). Chronic parental illness affects the relationship between parents with CMC and their children. For example, children may adopt roles that are inappropriate for their age and feel overwhelmed by the sheer presence of household chores and caregiving tasks. The assumption of an adult role by a child, referred to as parentification, is an example of a possibly modified familial interaction pattern (Faulkner and Davey 2002; Meijer et al. 2007; Pedersen and Revenson 2005). All the same, family relationships can remain a source of strength and should be acknowledged as a protective buffer for adolescent stress (Carr and Springer 2010).

Besides, adolescents’ gender and age may be associated with adolescent stress (Pedersen and Revenson 2005), but previous research delivered inconsistent results. Some studies found that girls have higher stress levels than boys (Sieh et al. 2010b; Welch et al. 1996). On the contrary, other studies reported no gender differences in child report of stress (Barkmann et al. 2007). Similarly, some studies suggested that older adolescents experience more stress than younger adolescents (Compas et al. 1994; Veach 1999), while other studies concluded that older adolescents experience less stress than younger adolescents (Faulkner and Davey 2002; Welch et al. 1996). It can be concluded that adolescents’ gender and age should be controlled for with regard to child report of stress.

Inspired by the FSI model, the relation of adolescent stress to specific parental CMC’s can be evaluated. Our sample concerns parents with a medical condition in the chronic stage, so we will examine in which regard the FSI model may be a useful explanatory framework apropos of child adjustment (adolescent stress) to chronic parental illness. To date, this has not been accomplished. According to the FSI model, chronic illnesses exhibit variability and without diagnostic differentiation, results may not apply to subsamples of children with a specific parental CMC. Therefore, the aim of this study is to investigate whether adolescents’ stress level varies while controlling for parental illness type. The first question is whether adolescent stress is directly influenced by illness type (disability, onset, course, and outcome). Our second question is whether the size and significance of the associations with parental depression, family functioning, and child demographics depend on illness type.

We expected that children of parents with a disabling CMC showed more stress than children of parents with a non-disabling CMC. Similarly, parental CMC’s with an acute onset were assumed to cause more stress in children than CMC’s with a gradual onset. In addition, children of parents with a progressive CMC were supposed to have a higher stress level than children of parents with a constant CMC. Children of parents with a possibly fatal CMC were expected to have a higher stress level than children of parents with a non-fatal CMC. Children of families with high family functioning were assumed to show less stress than children of families with low family functioning (Rolland 1987, 1999).

## Method

### Participants

We included children between 10 and 20 years of age who lived together with at least one parent with a CMC. Healthy partners, if applicable, also participated. Parental CMC was defined as a disease or a traumatic injury impairing health, involving one or more organ systems and lasting 6 months or longer (Brown et al. 2007; Livneh and Antonak 2005). Children with severe somatic or psychiatric disorders, which were assessed by way of parent interviews and child report, were not eligible for participation. Of 116 families showing interest in participation, only 17 families were not part of the final sample, resulting in a high participation rate (85.3 %). Eight families dropped out without indicating a reason. One family indicated to perceive participation as a burden. The remaining 7 families could not participate because their children were too old, too young, or disabled, or because no parent had a CMC. In one case, no data of the parent with CMC were available and the data for this family were deleted from the analyses, leading to a sample of 99 families including 158 adolescents and 82 healthy parents (Table 1). Most families consisted of married parents or couples living together. Fifteen families were counted as single parent household, four of which were characterized by a long distance relationship between the parents. In two families, both parents had a CMC and the data for the less disabled parent were excluded from analyses. All participants were Dutch. Most children followed lower vocational education. Over 40 % of the parents with CMC had finished higher vocational education or university. Parental CMC included multiple sclerosis (29.3 %), rheumatoid arthritis (19.2 %), brain damage (16.2 %), muscle disease (14.1 %), spinal cord injury (7.1 %), inflammatory bowel disease (6.1 %), Parkinson disease (5.1 %), and diabetes type I with physical complications (3.0 %). The mean time since diagnosis was 12.6 years and ranged between 7 months and 49 years.

**Table 1:** Demographic characteristics of adolescents, ill, and healthy parents in families with parental CMC

	*n* (%)	M (SD)
Adolescents	158	
Gender (female)	81 (51.3)	
Age		15.11 (2.32)
Highest educational level		
Primary education	27 (17.1)	
Lower vocational education	62 (39.2)	
Intermediate vocational education	23 (14.6)	
High school	38 (24.1)	
(Pre-)university education	7 (4.4)	
Ill parents	99	
Gender (female)	67 (67.7)	
Age		47.00 (5.43)
Highest educational level		
Primary/lower education	10 (10.1)	
Intermediate vocational education	29 (29.3)	
High school	11 (11.1)	
Pre-university or higher vocational education	30 (30.3)	
University	14 (14.1)	
Currently working	37 (37.4)	
Healthy parents	82	
Gender (female)	32 (31.7)	
Age		48.06 (5.74)
Highest educational level		
Primary/lower education	11 (12.2)	
Intermediate vocational education	26 (31.7)	
High school	6 (7.3)	
Pre-university or higher vocational education	25 (30.5)	
University	14 (14.1)	
Currently working	70 (85.4)	

### Measures

#### Illness Type

A medical doctor in our team documented illness type in a spread sheet in accordance with the FSI model (Rolland 1987; Stehouwer et al. 2010; World Health Organization 2001), see Table 2. When a parent had more than one CMC, the categorization of illness into illness type was based on the worst possible outcome and disability. Because of the small sample size, we decided to dichotomize the scales of the classification system with the following scores: non-disabling (0) versus disabling (1), gradual onset (0) versus acute onset (1), constant course (0) versus progressive course (1), non-fatal (0) versus potentially fatal (1).

**Table 2:** Number of parents with CMC as a function of illness type

		Non-disabling		Disabling	
		Gradual onset	Acute onset	Gradual onset	Acute onset
Non-fatal	Constant	1	1	0	23^a^
	Progressive	15^b^	0	18^c^	1
Potentially fatal	Constant	1	0	0	0
	Progressive	0	0	0	39^d^

Examples of CMC’s: ^a^multiple sclerosis, cystic fibrosis; ^b^rheumatoid arthritis, hereditary motor sensory neuropathy; ^c^stroke, spinal cord injury, traumatic brain injury; ^d^colitis ulcerosa. Disabling disease: expected problems with activities of daily living, and with communicative or cognitive activities like walking, dressing, and talking. Acute onset: onset of disease less than 1 h, diagnosis easily made. Progressive course: disease increasing in severity. Possibly fatal: possibility of death or shortened life span

#### Adolescent Stress

Adolescents filled in the Dutch Stress Questionnaire for Children (Hartong et al. 2003), a reliable self-report measure to determine global psychological stress (17 items; 4-point Likert scale from 1 = *not true for me at all* to 4 = *completely true for me*). In this study, Cronbach’s alpha was *α* = 0.87.

#### Depressive Symptoms in Ill Parent’s 

Depressive symptoms of parents with CMC, also referred to as parental depression, were determined with the Beck Depression Inventory (21 items; 4-point scale from 0 = *I do not feel like a failure* to 3 = *I feel I am a complete failure as a person, α* = 0.87) (Beck et al. 1961; Visser et al. 2006; Yin and Fan 2000).

#### Family Functioning

Three scales were used to assess family functioning. The Interactional Problem Solving Inventory (IPSI) measuring perceived quality of marital relationship from Lange (1983) was filled in by both parents. Only 10 single parents with CMC did not fill in the IPSI. Where applicable, we calculated dyadic scores reflecting the quality of marital relationship for each family (17 items; 5-point Likert scale from 1 = *exactly applicable to me/my partner* to 5 = *absolutely not applicable to me/my partner*; *α* = 0.85). To assess the quality of parent-child interaction, both parents completed the Parent-Child Interaction Questionnaire Revised from Lange (2001). Average scores were calculated to generate one parent-child interaction score for each family (21 items; 5-point Likert scale; 1 = *completely inapplicable,* 4 = *exactly applicable*; *α* = 0.87). In addition, adolescents filled in three scales (communication, confidence, and alienation) of the Inventory of Parent and Peer Attachment (Nada Raja et al. 1992; Reitz 2004) about the father and the mother, and average scores were calculated to measure overall parent attachment (12 items; 4-point Likert scale from 1 = *almost never or never* to 4 = *almost always or always*; *α* = 0.88).

#### Adolescents’ Gender and Age

Gender was scored as male (0) and female (1). We used the exact age with two decimals.

### Procedure

Families with parental CMC were recruited through general health practitioners, health organizations, rehabilitation and community centers, hospitals, schools, and public places (e.g., libraries) across the Netherlands. Collaborating staff was instructed by the project manager and posted brochures and posters in waiting rooms and public spaces. Some of them also provided additional information and invited potential participants to take part in this study. Families had to contact the researchers by e-mail or phone to show their interest in participation. Research assistants visited the families at home to implement several questionnaires. Both parents and adolescents provided active informed consent. The ethical commission of the research institute of Child Development and Education of the University of Amsterdam approved this study.

### Data Analyses

The research questions were answered by means of multilevel regression analyses which accounted for the fact that children within families have more similarities than children between families (Snijders and Bosker 1999). We used linear mixed modeling of SPSS, version 20.0. Adolescents acted as Level 1, while the family acted as Level 2. The nesting structure of adolescents and chronically ill parents is illustrated in Table 3.

**Table 3:** Nesting structure of adolescents per marital status or living condition

	Number of adolescents within families				
	1	2	3	4	Total
Single parent home	10	4	1	0	15
Parents married/ living together	41	34	8	1	84
Total	51	38	9	1	99

Using G*Power (Buchner et al. 2009), we found that the power of this study was 0.89, while correcting for nested data with the expectation of equal sample sizes in each group. A power value that large means that it was probable that we would find a statistically significant result when such a result actually exists. Due to a small sample size within many cells, no analysis could be performed including all illness types at once. Four analyses of illness type were conducted separately and included all other predictors in each analysis. In the first analysis (Model 1), the influence of disability was examined. Model 2 concerned the influence of illness onset (gradual versus acute). Model 3 included constant illnesses versus progressive illnesses. Model 4 examined non-fatal CMC’s versus potentially fatal CMC’s. We report the Akaike information criterion as a measure of the relative goodness of model fit. To illustrate comparable estimates, all independent variables were standardized.

Only 0.9 % of the data from children and 3.5 % of the data from parents were missing throughout the dataset. Data were missing completely at random (Little 1988), so we used expectation maximization to substitute missing values.

## Results

Children were distributed among the following categories of parental illness type: 132 cases of disability versus 26 cases of no disability, 117 cases of gradual onset versus 41 cases of acute onset, 44 cases of constant course versus 114 cases of progressive course, and 92 cases of non-fatal outcome versus 66 cases of possibly fatal outcome.

The level of adolescent stress was comparable to that of children 3 years after parental stroke (Sieh et al. 2010b). Half of the ill parents (50.5 %) displayed scores that indicated mild (29.3 %), moderate (10.1 %), or severe depression (11.1 %). The mean quality of marital relationship was under the cut-off score (68.5 points) and 69.5 % of the parents scored below the cut-off score, indicating relatively poor marital functioning. The average scores for quality of parent-child interaction were very close to those of the normal population (mean = 88.82, *SD* = 6.59; Lange 2001), see Table 4.

**Table 4:** Descriptive statistics for adolescent stress, parental depression, quality of marital relationship, quality of parent-child interaction and quality of parent attachment

	Mean (*SD*)	Range
Adolescent stress	34.66 (8.07)	17.0-63.0
Parental depression	12.19 (7.70)	1.0-34.0
Quality of marital relationship	62.01 (9.96)	34.0-78.0
Quality of parent-child interaction	88.99 (10.69)	49.0-104.0
Quality of parent attachment	38.89 (5.59)	15.5-48.0

All independent variables (parental depression; quality of marital relationship, parent-child interaction, and attachment; adolescent gender and age) had a significant correlation with adolescent stress, but only quality of parent attachment showed a large correlation and all other correlations with adolescent stress ranged between small and medium (Cohen, 1992), see Table 5. Most correlations between the predictors were significant, one of which was large in size, indicating that higher quality of marital relationship was strongly associated with lower parental depression scores. The size of the other correlations between family functioning variables and parental depression, and within family functioning variables was medium. Adolescents’ gender and age showed three small correlations, that is, girls and older children comparatively reported more stress and older children reported a lower quality of parent attachment.

**Table 5:** Correlations between Adolescent Stress, Parental Depression, Family Functioning, and Adolescents’ Gender and Age

	1	2	3	4	5	6
1 Adolescent stress	-					
2 Parental depression	0.24**	-				
3 Quality of marital relationship	-0.20*	-0.67**	-			
4 Quality of parent-child interaction	-0.30**	-0.32**	0.42**	-		
5 Quality of parent attachment	-0.56**	-0.31**	0.36**	0.29**	-	
6 Adolescents’ gender	0.19*	-0.04	0.01	0.05	-0.03	-
7 Adolescents’ age	0.25**	-0.04	-0.04	-0.10	-0.25**	0.01

*p < 0.05. **p < 0.01

An empty model was estimated, with only the random intercept and the family as the grouping variable (Table 6). The intra-class correlation coefficient (ICC = 0.29) and the deviance ratio [*χ*²(1) = 4.47, *p* < 0.05] indicated that a model with a random intercept fitted the data better than a model that did not allow for random variability. The ICC can be interpreted as such that two random children in the same random family shared 29 % of the variability.

**Table 6:** Model specifications of multilevel analyses, including 95 % Confidence Intervals [CI] of fixed effects

	Empty model		Model 1		Model 2		Model 3		Model 4	
	Estimate	S.E.	Estimate	S.E.	Estimate	S.E.	Estimate	S.E.	Estimate	S.E.
Intercept	34.61***	0.72	34.47***	1.40	33.47***	0.81	32.75***	1.24	33.00***	0.92
Disability			-1.69 [-4.60, 1.21]	1.46						
Illness onset					-1.44 [-4.03, 1.13]	1.30				
Illness course							0.46 [-2.12, 3.03]	1.29		
Illness outcome									0.23 [-2.02, 2.47]	1.13
Parental depression			1.17 [-0.31, 2.65]	0.74	1.11 [-38, 2.61]	0.75	1.08 [-0.44, 2.59]	0.76	1.11 [-38, 2.61]	0.75
Quality of marital relationship			1.24 [-0.32, 2.80]	0.78	1.25 [-0.32, 2.82]	0.79	1.25 [-0.21, 2.83]	0.79	1.25 [-0.32, 2.82]	0.79
Quality of parent-child interaction			-1.38* [-2.62, -0.14]	0.62	-1.44* [-2.70, -0.19]	0.63	-1.48* [-2.76, -0.20]	0.64	-1.44 [-2.69, -0.19]	0.63
Quality of parent attachment			-3.97*** [-5.13, -2.80]	0.59	-3.97*** [-5.13, -2.79]	0.59	-3.96*** [-5.13, -2.79]	0.59	-3.97*** [-5.13, -2.79]	0.59
Adolescents’ gender			3.13** [1.27, 5.16]	1.03	3.06** [1.03, 5.11]	1.03	3.08** [1.51, 5.42]	1.03	3.06** [1.03, 5.11]	1.03
Adolescents’ age			0.94 [-0.12, 2.00]	0.54	0.93 [-0.13, 2.00]	0.54	0.95* [-0.12, 2.03]	0.54	0.93 [-0.13, 2.00]	0.54
Level 1 variance	46.07		34.94		34.73		34.84		34.92	
Level 2 variance	19.86		6.35		6.62		6.86		6.78	
AIC	1105.74		1021.50		1021.83		1022.97		1023.33	
Explained variance (Level 1)			37.4 %		37.3 %		36.8 %		36.8 %	
Explained variance (Level 2)			43.4 %		44.7 %		43.4 %		43.5 %	

*AIC* Akaike Information Criterion. Explained variance at Level 2 was calculated assuming that families have two children on average.*p < 0.05. **p < 0.01. ***p < 0.001

First, Model 1 was tested while controlling for the presence of disability. Quality of parent-child interaction and parent attachment, and adolescents’ gender were significantly linked to adolescents’ stress level, see Table 6. Disability of ill parents was not related to adolescent stress. Model 2 concerned the illness onset characterized as either gradual or acute. Estimates for quality of parent-child interaction and parent attachment, and adolescents’ gender were statistically significant. Illness onset was not associated with adolescent stress. Model 3 controlled for constant and progressive CMC’s. Quality of parent-child interaction and parent attachment, and adolescents’ gender displayed significant associations with adolescent stress. Illness course was unrelated to adolescent stress. Model 4 included the outcome of the illness (non-fatal versus possibly fatal). Again, quality of parent-child interaction and parent attachment, and adolescents’ gender had significant relationships with adolescent stress. Illness outcome was not linked to adolescent stress.

In sum, the results demonstrated that independent of illness type, higher quality of parent-child relationships and adolescents’ female gender were connected to elevated adolescent stress. The Akaike Information Criterion of Model 1 to Model 4 showed that the model fit improved compared to the empty model. The four models explained approximately 37 % of the variance in adolescent stress between individuals and 43-44 % of the variance in adolescent stress between families.

## Discussion

This study evaluated the predictive power of illness type, the ill parent’s depressive symptoms, family functioning, and adolescents’ gender and age on child report of stress in a sample of parents with a medical condition in the chronic stage. First, illness type in the chronic stage of parental illness did not appear to affect adolescent stress scores. Second, the size and significance of the estimates for parental depression, family functioning and child demographics did not depend on illness type. Hence, the results cannot confirm the expectation based on the FSI model suggesting a significant role of illness type with respect to child report of stress. Contrary to our hypotheses, not illness type but mainly family functioning and adolescents’ gender were directly related to adolescent stress. All variances explained by the models were moderate, meaning that we identified important risk and protective factors for adolescent stress at the individual level (i.e., quality of parent attachment and adolescent gender) and at family level (i.e., quality of parent-child interaction). In support of the FSI model, strong evidence was found that the parent-child relationship is a crucial determinant of adolescent stress.

Unexpectedly, the relationship between parental depression and adolescent stress was not significant in regression analyses. On the contrary, the empirical and theoretical basis suggests that depressive symptoms of ill parents often emerge over time (Sieh et al. 2010b), eminently in more debilitating and progressive conditions. In accordance, Rolland (1987; 1999) stated that disabling and possibly fatal diseases have the strongest impact on family functioning. We did not find support for this assumption and can only present a small to medium positive correlation between parental depression and adolescent stress. Possibly, other mediating variables (e.g., parenting and coping variables) are involved in the relationship between ill parents’ depressive symptoms and adolescent stress (D’Onofrio and Lahey 2010; Sieh et al. 2010b).

Similarly, high quality of marital relationship was merely correlated with elevated adolescent stress. However, no significant relationship was found between marital functioning and adolescent stress when we controlled for illness type, parent-child interaction, parent attachment, and demographics. Research is inconsistent about the influence of quality of marital relationship on adolescents’ functioning (Compas et al. 1994, 1996; Faulkner and Davey 2002; Sieh et al. 2010b; Veach 1999). In our sample, marital functioning was poor, resulting in lower variance, which may obstruct significant results. A longitudinal study on families of stroke patients showed that marital functioning progressively decreased under the level of adequate functioning. Marital functioning during 3 years post-stoke was just incidentally associated with long-term stress in adolescents (Sieh et al. 2010b). A possible explication for inconsistent findings in the field is that marital functioning and child adjustment are bidirectional and reinforce each other through a mediating variable such as parenting and parent attachment (D’Onofrio and Lahey 2010).

High quality of parent-child interaction and especially high quality of parent attachment were associated with lower levels of adolescent stress, pointing to a potential protective mechanism in child adjustment to parental CMC. Notably, the estimates for parent attachment were the highest. As yet, previous research has attested the paramount importance of family functioning in child adjustment to parental CMC (Carr and Springer 2010; Hocking and Lochman 2005; Korneluk and Lee 1998; Sieh et al. 2010a). Our study extends previous knowledge, affirming that especially variables evaluating the parent-child relationship show high predictive power for adolescent stress. This suggests that rehabilitation staff can be recommended to give attention to the parenting role, how the parenting role may change as a result of CMC, and how parental CMC affects the parent-child relationship. For example, parents may need reassurance when they do a good job. They could also benefit from advice about how to talk to children about their condition and its impact on family life. In addition, the results substantiate that the parent-child relationship should be evaluated by both parents and adolescents who may evaluate the mutual relationship differently (Brumariu and Kerns 2010; Korneluk and Lee 1998; Meijer et al. 2007).

Moreover, girls appear to be more susceptible to stress than boys, which is in line with most research in the field (Sieh et al. 2010a; b; Verhaeghe et al. 2005). The estimate for gender was highly significant, bringing into view that girls have different sensitivities than boys. It may be hypothesized that girls could benefit from stress management more than boys. Lastly, adolescents’ age was unrelated to child report of stress in the regression analysis and we found a small positive correlation. Previous research is inconsistent about the influence of adolescents’ age on adolescents’ well-being (Faulkner and Davey 2002; Pedersen and Revenson 2005; Sieh et al. 2010a). More research is needed for distinct conclusions regarding the effects of age on adolescent stress in this specific group.

This study had some limitations. Our sample size was small for the amount of cases per illness type except in Model 1 (including disability). A related restraint is that we had to dichotomize the scales of illness type and could not account for progressive illness course or fatal outcomes, potentially altering the results. For example, illness outcome may have been unrelated to adolescent stress due to the omission of illness fatality. Similarly, because of the sample size, we had to ignore the interactive effects of one versus two parents with a disability, and one CMC versus more than one CMC. Moreover, it should be borne in mind that we examined CMC in a sample of Western culture. The results could have looked differently using data from a sample of non-Western culture because of distinctive cultural views and practices affecting health and health behaviors (Carr and Springer 2010).

An important strength of this study lies in the inspiration from the FSI model in which medical information is combined with information about family functioning of the parent and adolescent. In addition, this study recognized factors that emerged as influential predictors in previous research, including reports of the parent-child relationship from parents and adolescents. As recommended (D’Onofrio and Lahey 2010), we took into consideration family characteristics at multiple levels (demographic, psychological, and relational variables from several informants). Finally, the use of multilevel analyses is an advancement as grouping according to families led to a considerable similarity between adolescents within the same family.

Our study did not aim to generate specific recommendations for the clinical practice, but possible interventions should not go unmentioned. In an extensive review, Weihs et al. (2002) stratified studies by specific CMC’s and formulated three general goals for interventions for families affected by CMC. Recommendations include helping families cope with illness-related stressors, mobilizing the family support system, and minimizing interpersonal hostility and adverse effects of illness-related trauma. Carr and Springer (2010) reviewed research on families and health between 2000 and 2009. In line with our findings stressing the importance of family relationships, Carr and Springer identify interpersonal relationships as potentially modifiable factors. Interventions should aim to encourage healthy relationships through parent education, conflict and stress management, communicative training, and health promotion. For example, cognitive-behavioral stress management training has shown to benefit mental health, social interaction, and family functioning in adolescents with parental CMC (Keypour et al. 2011).

Future studies should incorporate all elements of the FSI model, taking into account the stage of an illness consisting of crisis, chronic stage, and terminal stage (Rolland 1987, 1999). Stages of individual family members and family life cycles also need to be considered. Our data were collected in the chronic stage, making it impossible to compare the stress level in different illness stages. With a larger and more diverse illness sample, the FSI model can be fully applied and the hypotheses can be formulated in more complex ways. Notably, the transitions between time stages are crucial in relation to the other illness types. For instance, the stressors associated with the crisis stage of a less severe condition could easily generate more strain on a family system than the chronic phase of a more serious condition. Similarly, the hypothesis that acute onset CMC is associated with more stress in adolescents than a gradual onset CMC could depend on the time stage in which families are investigated. In case of parental stroke (acute onset) relative to multiple sclerosis (gradual onset), the hypothesis may initially hold. Yet, if the measurement occurred several years later, families dealing with a stroke would show considerable improvement over time (Visser-Meily et al., 2009), potentially decreasing the experience of stress (Sieh et al. 2010b). Besides, the presence of disability depends on the stage of illness. To illustrate, multiple sclerosis can be non-disabling in the beginning and cause severe disability a decade later. These complex relationships between illness type and illness stage could lead to interesting results from forthcoming longitudinal studies (Rolland 1987, 1999; Rolland and Williams 2005). Further, studies could focus on the mediating and moderating role of stress and family characteristics in the development of problem behavior in adolescents. D’Onofrio and Lahey (2010) have conducted a decade review on biosocial studies focusing on family processes. They emphasize the need to be thoughtful of bidirectional influences among family members and the wider social and biological system, embodying interactions between environmental and biological factors (e.g., stress hormones like cortisol) over time.

Lastly, we investigated medical and psychological factors, whereas theories and models are usually developed from a specific perspective, for example, a medical perspective. To increase our understanding of the complex FSI model, it is necessary to better understand family relationships and the interplay between all components of the model. This can be accomplished by means of transdisciplinary theories and models, for example, the social model of disability (Tate and Pledger 2003). According to that model, the perceived stressfulness does not necessarily result from the parent’s physical disability but rather from interpersonal experiences and environmental mismatches that functional limitations can precipitate.

In conclusion, this study increases our understanding of the FSI model and provides insight into protective and risk factors for adolescent stress in the chronic stage of parental medical condition. We found some support for the FSI model in the sense that family functioning is associated with adolescent stress, but this association does not seem to depend on parental illness type in the chronic stage. It is valuable to incorporate interpersonal family variables and adolescents’ gender in predictive models of adolescent stress. Rehabilitation staff is recommended to consider how parental CMC affects the parent-child relationship and how family relationships can be boosted, offering interventions such as stress and conflict management.
